# Do pre-hospital anaesthesiologists reliably predict mortality using the NACA severity score? A retrospective cohort study

**DOI:** 10.1111/aas.12208

**Published:** 2013-10-17

**Authors:** L RAATINIEMI, K MIKKELSEN, K FREDRIKSEN, T WISBORG

**Affiliations:** 1 Department of Anaesthesiology and Intensive Care, Hammerfest Hospital, Finnmark Health TrustHammerfest; 2 Division of Emergency Medical Services, University Hospital of North Norway TromsøNorway; 3 Anaesthesia and Critical Care Research Group, Department of Clinical Medicine, Faculty of Health Sciences, University of TromsøTromsø, Norway; 4 Norwegian Trauma Competency Service, Oslo University HospitalOslo, Norway; 5 Department of Anaesthesiology, Lapland Central HospitalRovaniemi, Finland

## Abstract

**Introduction:**

The National Advisory Committee on Aeronautics' (NACA) severity score is widely used in pre-hospital emergency medicine to grade the severity of illness or trauma in patient groups but is scarcely validated. The aim of this study was to assess the score's ability to predict mortality and need for advanced in-hospital interventions in a cohort from one anaesthesiologist-manned helicopter service in Northern Norway.

**Methods:**

All missions completed by one helicopter service during January 1999 to December 2009 were reviewed. One thousand eight hundred forty-one patients were assessed by the NACA score. Pre-hospital and in-hospital interventions were collected from patient records. The relationship between NACA score and the outcome measures was assessed using receiver operating characteristic (ROC) curves.

**Results:**

A total of 1533 patients were included in the analysis; uninjured and dead victims were excluded per protocol. Overall mortality rate of the patients with NACA score 1–6 was 5.2%. Trauma patients with NACA score 1–6 had overall mortality rate of 1.9% (12/625) and non-trauma patients 7.4% (67/908). The NACA score's ability to predict mortality was assessed by using ROC area under curve (AUC) and was 0.86 for all, 0.82 for non-trauma and 0.98 for trauma patients. The NACA score's ability to predict a need for respiratory therapy within 24 h revealed an AUC of 0.90 for all patients combined.

**Conclusion:**

The NACA score had good discrimination for predicting mortality and need for respiratory therapy. It is thus useful as a tool to measure overall severity of the patient population in this kind of emergency medicine system.

The National Advisory Committee of Aeronautics (NACA) severity score has gained widespread acceptance as a means of describing disease or injury severity in patients treated by emergency medical services (EMS). It is an eight level subjective severity score (Table 1). The NACA score was one of the earliest severity scoring systems in trauma and was primarily used for scoring 24 h after admission in hospital.^1^,^2^ Because of this, the NACA score was not applied for pre-hospital purposes. In 1980, Tryba et al. modified the NACA score to include both surgical and medical conditions at the time of handover from EMS to hospital^3^ and thus made the score suitable for severity assessment by pre-hospital services. The NACA score is mainly used for characterisation of groups of patients and for rapid descriptions of the severity of individual patients, but it has never been intended for prognostication or triage for individual patients. Despite its widespread use, the NACA score has been validated in only a few of the various EMS where it is in use. In one study from a Central European EMS system, Weiss et al. showed that the NACA score correlates well with mortality and morbidity.^2^ In another study, done by Bonatti et al., the NACA score seemed to be an independent predictor of survival rate at 30 days.^4^

**Table 1:** The severity scoring used to classify injury or illness severity in the Norwegian National Air Ambulance Service as originally described by the National Advisory Committee on Aeronautics (NACA).^4^

NACA O	No injury or disease
NACA l	Injuries/diseases without any need for acute physicians care
NACA 2	Injuries/diseases requiring examination and therapy by a physician, but hospital admission is not indicated
NACA 3	Injuries/diseases without acute threat to life but requiring hospital admission
NACA 4	Injuries/diseases that can possibly lead to deterioration of vital signs
NACA 5	Injuries/diseases with acute threat to life
NACA 6	Injuries/diseases transported after successful resuscitation of vital signs
NACA 7	Lethal injuries or diseases (with or without resuscitation attempts)

The NACA score has been used in the Norwegian air ambulance system since the 1980s and is used to compare the patient characteristics between different air ambulance bases. It is used for both trauma and non-trauma patients. The NACA score has not been validated in Norway, and its ability to predict mortality and in-hospital interventions in Norwegian air ambulance patients is not known. Ideally, a severity score should predict reasonably the risk of mortality and preferably also the need for advanced interventions at a group level.

We aimed to assess the NACA score's ability to predict mortality (primary outcome measure) and need for advanced in-hospital interventions (secondary outcome measure) in a retrospective cohort study from one single anaesthesiologist-manned rescue helicopter base in Northern Norway.

## Material and methods

The Norwegian Air Ambulance Service is a nationwide system served by helicopter and fixed wing aircraft bases.^5^ The Royal Norwegian Air Force's anaesthesiologist-manned 330 Squadron is a dedicated search and rescue (SAR) helicopter service and also contributes regularly to the national Air Ambulance system. We reviewed all missions completed by the SAR base at Banak, Northern Norway, during the period 1 January 1999 to 31 December 2009. We included all patients that had been treated by the service and assessed using the NACA score. Pre-hospital data (patient ID, date of mission, diagnosis, NACA score, pre-hospital interventions and the institution to which the patient was admitted) were collected from the service's electronic patient record.

The hospitals in Hammerfest, Kirkenes and Tromsø receive patients from the service. Relevant in-hospital data from the hospital records of the patients were recorded. Ventilatory support was defined as the institution or continuation of any form of positive pressure ventilation either via endotracheal intubation or non-invasive ventilatory support during the first 24 h after admission. Haemostatic emergency surgery was assessed at two levels of definitions: (1) defined as haemostatic packing of the abdomen or pelvis, or thoracotomy exceeding tube thoracostomy, and (2) the earlier definition plus tube thoracostomy and/or emergency orthopaedic procedures performed within 24 h.

Thirty-day mortality was assessed using the hospitals' medical records based on The National Population Register. Norwegian patients from the catchment area of the hospitals discharged before 30 days after admission have their medical records updated with survival data from The National Population Register, while persons living outside the region were lost to follow-up with regards to mortality. Patients without information on 30-day mortality were considered as survivors if they were discharged to their home directly, even if a follow-up consultation was planned.

Data collection was performed by two experienced consultant anaesthesiologists, with more than 4 years of experience in pre-hospital emergency medicine. The relationship between the NACA score and the outcome measures was assessed using receiver operating characteristic (ROC) curves. With this test, the true positive rate (sensitivity) is plotted against the false-positive rate (1 – specificity) to receive a graphic estimate of the test or scoring system performance as the area under the curve (AUC).^6^–^9^ We regarded an AUC of more than 0.8 as a good and an AUC of more than 0.95 as excellent predictor of outcome.

### Statistics

Data were entered into an Excel spread sheet and subsequently transformed to SPSS (IBM Corp., Armonk, NY, USA) for ROC calculations. The Chi-square test was used for comparisons of proportions (MedCalc software v. 11.1, MedCalc Sofware bvba, Ostend, Belgium).

### Ethics

The study was approved by the Regional Committee for Medical and Health Research Ethics of Northern Norway (2010/3263-9).

## Results

**Figure 1:**
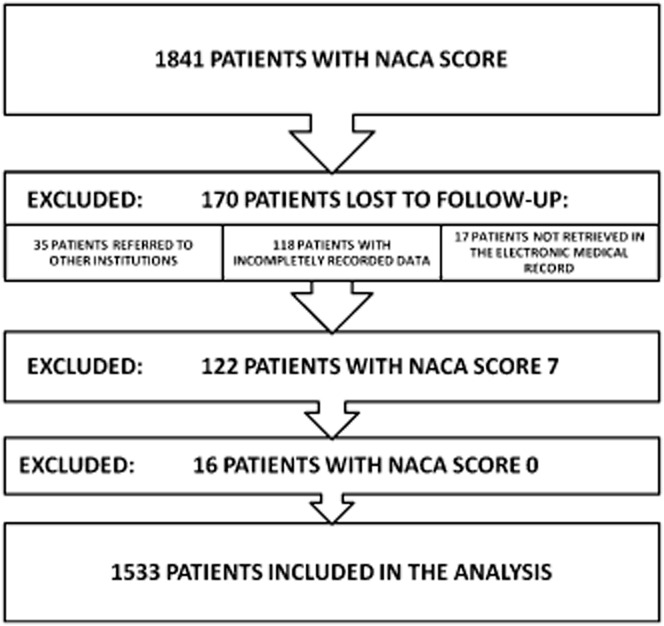
Patients excluded and included in the analysis. NACA, National Advisory Committee of Aeronautics.

**Figure 2:**
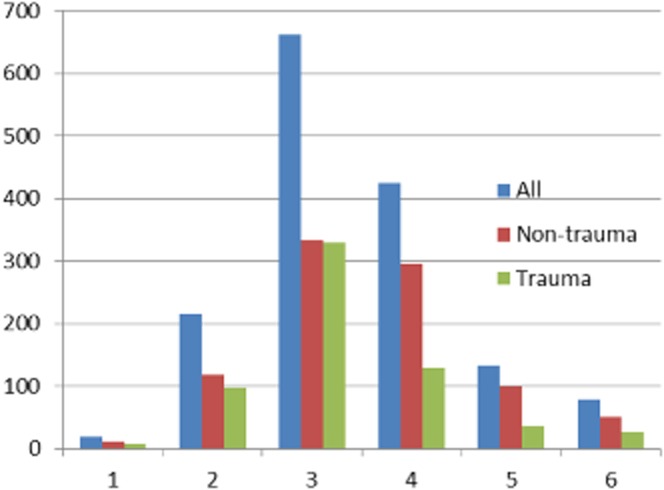
Distribution of National Advisory Committee of Aeronautics scores in the included patients divided in trauma and non-trauma conditions (*n* = 1533).

During the 11-year period, 1841 patients were evaluated on scene and scored by the air ambulance anaesthesiologist using the NACA score. Of these, 122 patients succumbing before scene arrival or during transportation were scored as NACA 7 per definition and subsequently excluded from the study. Sixteen patients were not injured (rescue missions) and were designated as NACA 0. One hundred and seventy patients were lost to follow-up; 35 of these were referred to other institutions in Norway or abroad during the first 30 days after treatment, the data of 118 patients was incompletely recorded, and 17 of the patients were not retrieved in the electronic medical records at all. The majority of these patients had a NACA score of 3 or 4 (Table 2). Thus, a total of 1533 out of 1703 patients with NACA score 1–6 were included for further analysis of mortality and interventions (Figs 1 and 2).

**Table 2:** Patients lost to follow-up with National Advisory Committee on Aeronautics (NACA) scores

	Incomplete data	Transferred to other hospital	Not retrieved in medical records	Total
NACA 1	5	0	0	5
NACA 2	22	1	3	26
NACA 3	46	13	4	63
NACA 4	31	16	4	51
NACA 5	9	3	5	17
NACA 6	5	2	1	8

Nine hundred and eight patients had a medical diagnosis, while 625 (41 %) suffered from trauma. The median age was 50 years (interquartile range 32–66 years) in the medical group and 34 years (interquartile range 20–50 years) in the trauma group. Males comprised 59.5% of the medical cases and 74.6 % of the trauma cases. The median NACA score was 3, with an interquartile range of 3–4; the mean NACA score was 3.44 (*n* = 1533).

### Mortality

To investigate the relation between NACA severity score and patient mortality, we assessed the 30-day mortality for all NACA score categories. The overall 30-day mortality in the material was 5.2% (79/1533). The data for all NACA scores are shown in Table 3. The non-trauma patients had a significantly higher overall mortality (7.4% vs. 1.9%, *P* < 0.0001).

**Table 3:** Thirty-day mortality, sensitivity, specificity, PPV and NPV for different NACA scores

Patient category	n	30-day mortality (n)	Mortality percent (95 % CI)	Sensitivity	Specificity	PPV	NPV
Trauma patients	625	12	1.9% (1.1–3.3)				
NACA 1	8	0	0.0% (0.0–32.4)	1.00	1.00	0.02	–
NACA 2	97	0	0.0% (0.0–3.8)	1.00	0.01	0.02	1.00
NACA 3	329	0	0.0% (0.0–1.2)	1.00	0.17	0.02	1.00
NACA 4	129	0	0.0% (0.0–2.9)	1.00	0.71	0.06	1.00
NACA 5	35	1	2.9% (0.5–14.5)	1.00	0.92	0.19	1.00
NACA 6	27	11	40.7% (24.5–59.3)	0.92	0.98	0.41	1.00
Non-trauma patients	908	67	7.4% (5.9–9.3)				
NACA 1	11	0	0.0% (0.0–25.9)	1.00	0.00	0.07	–
NACA 2	118	3	2.5% (0.9–7.2)	1.00	0.01	0.07	1.00
NACA 3	333	4	1.2% (0.5–3.0)	0.96	0.15	0.08	0.98
NACA 4	296	17	5.7% (3.6–9.0)	0.90	0.54	0.13	0.98
NACA 5	99	19	19.2% (12.6–28.0)	0.64	0.87	0.29	0.97
NACA 6	51	24	47.1% (34.1–60.5)	0.36	0.97	0.47	0.95

CI, confidence interval; NACA, National Advisory Committee of Aeronautics; NPV, negative predictive value; PPV, positive predictive value.

**Figure 3:**
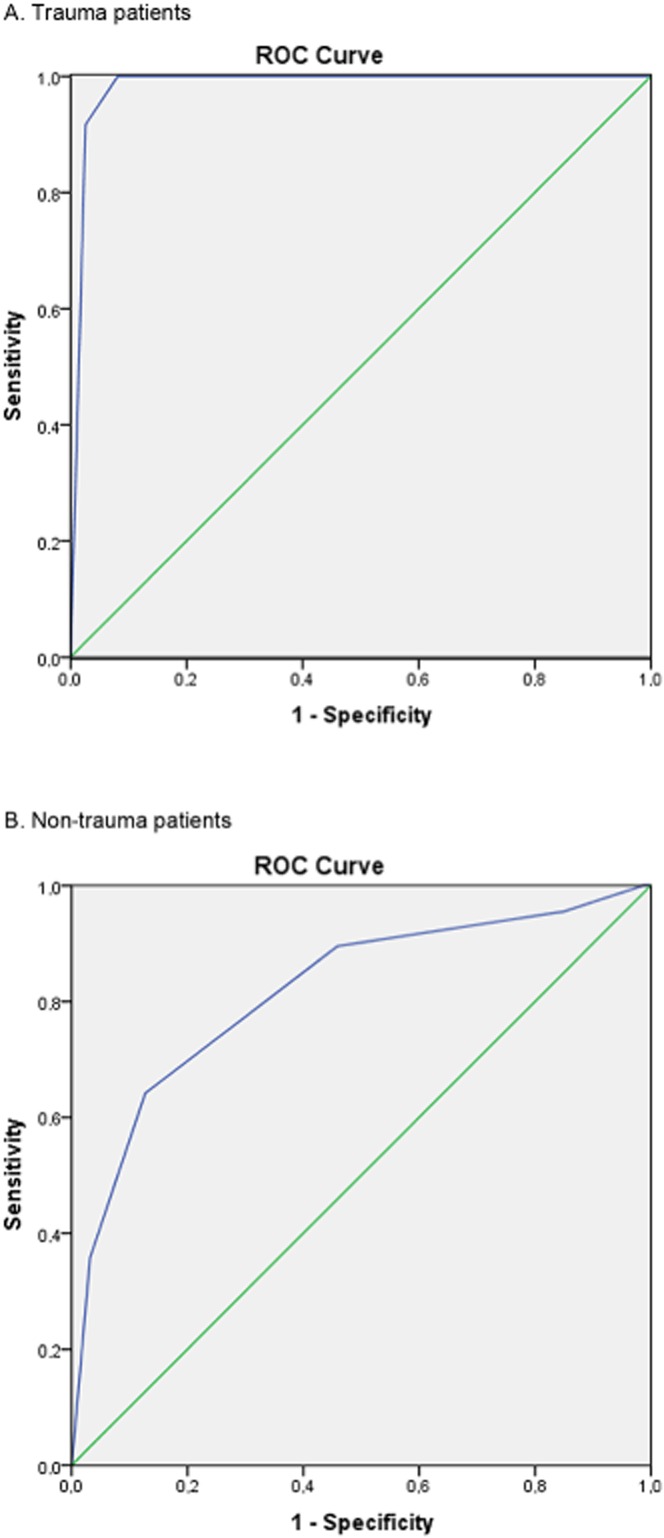
Receiver operating curves depicting the National Advisory Committee of Aeronautics score as a predictor of 30-day mortality in two cohorts of (A) trauma patients and (B) non-trauma patients from one Norwegian rescue helicopter base. Area under curve (AUC) for trauma patients was 0.98 [95% confidence interval (CI) 0.97–1.00], and AUC for non-trauma patients was 0.82 (95% CI 0.76–0.88). The abscissa is 1 – specificity (false-positive); the ordinate is sensitivity.

The NACA score's sensitivity and specificity for predicting death was plotted in a ROC curve in order to assess the score's ability to predict mortality. (Table 4) The ability to predict mortality was excellent for trauma patients, with an AUC of 0.98, clearly higher than the AUC for all patients (0.86) and for non-trauma patients (0.82). ROC curves for mortality in the patient groups are shown in Fig. 3. If a cut-off level of 5 or higher was chosen, the score sensitivity for 30-day mortality was 1.00 in trauma patients and the specificity 0.92. This gave a positive predictive value (PPV) of 0.19, and the negative predictive value (NPV) was 1.00 in this subgroup. With the same cut-off level of 5, the sensitivity was much lower in the non-trauma patients (0.64). The corresponding specificity was 0.87, PPV 0.29 and NPV 0.97.

**Table 4:** The ability of the pre-hospital severity assessment with the NACA score to predict mortality and advanced interventions in hospital analysed by ROC AUC with 95% CI

All patients n = 1533	Subgroups:	No. with end point	ROC AUC	95% CI
All patients				
Mortality		79	0.86	0.81–0.91
Ventilatory support		137	0.90	0.87–0.93
	Non-trauma patients, *n* = 908			
	Mortality	67	0.82	0.76–0.88
	Ventilatory support	90	0.88	0.84–0.92
	Trauma patients, *n* = 625			
	Mortality	12	0.98	0.97–1.00
	Haemostatic emergency laparotomy/thoracotomy	5	0.94	0.87–1.00
	Haemostatic emergency surgery including tube thoracostomy and emergency orthopaedics	36	0.76	0.68–0.85
	Ventilatory support	47	0.94	0.90–0.97

For detailed definitions of hemostatic emergency surgery, see Materials and methods section.

CI, confidence interval; NACA, National Advisory Committee of Aeronautics; ROC AUC, receiver operator characteristic area under curve.

Seven non-trauma patients with low NACA scores (2 and 3) died within 30 days after admission to hospital. Five of these patients obviously had a more severe condition than expected during the pre-hospital treatment when assessed retrospectively. Two patients had cancer and died from the malignant disease during the month after admission, but their clinical condition was not evaluated as serious in the pre-hospital phase.

### Advanced in-hospital interventions

The ROC characteristics for the NACA score's prediction of three different secondary outcome measures after admission were calculated. The results are shown with AUC and 95% confidence intervals in Table 4.

Haemostatic emergency surgery defined as haemostatic packing of the abdomen or pelvis, or thoracotomy was performed in only 0.8% (5/625) of the trauma patients. The AUC for the NACA score as a predictor of emergency surgery was 0.94 (95% CI 0.87–1.0). When a broader definition of haemostatic emergency surgery was applied (*n* = 36), which also included tube thoracostomy and emergency orthopaedic procedures, the AUC for the NACA score as a predictor was 0.76 (95% CI 0.68–0.85).

Ventilatory support during the first 24 h after admission was received by 8.9% (137/1533) of the patients. The AUC was 0.90 for all the patients, with no significant differences in frequency between trauma patients and non-trauma patients (*P* = 0.128).

## Discussion

This study shows that the pre-hospital NACA score is a reliable predictor of both mortality and need for advanced interventions in-hospital in a single helicopter base cohort through 11 years. Our results thus confirm the two previous European studies.^2^,^4^

The mortality was higher for non-trauma patients than trauma patients in the study population. This might be expected if the severely ill non-trauma patients have a higher age and more co-existing diseases than the trauma patients. Our study consisted of both medical and trauma patients contrary to the study of Weiss et al. that consisted of trauma patients only.^2^ The mortality rate of the trauma patients in our study material was 1.9%, which is lower than that in the study by Weiss et al. (6.8%). The overall mortality in our material was lower compared with the study of Bonatti et al. (5.2% vs. 12.1%).^4^ A possible explanation is differences in patient demography and thus a low median NACA score in our study material.

The mortality for patients with a NACA score of 5 was 14.9%, which is similar to the results published by Weiss et al.^2^ (12.2 %) and Bonatti et al.^4^ (14.4 %). Interestingly, our population had a much lower mortality in the NACA 6 group than the cited studies. This can be a result of a higher pre-hospital mortality for the most severely injured or diseased patients in our rural population. Indeed, it has earlier been reported that the time distribution of trauma deaths in the county of Finnmark differs from urban populations, in that a far higher fraction of the severe trauma cases die before they arrive in hospital.^10^ This observation may at least to some extent explain our finding.

Another important indicator of severity is whether the patients required advanced life-saving therapy shortly after admission and whether they needed admission to the intensive care unit (ICU). We found that the NACA category predicted a need for ventilatory support in the present study. This is similar to the findings of the study by Weiss and colleagues, who found a strong correlation between the score and admission to intensive care but a weaker association with length of hospital stay or stay in the ICU.^2^ The need for haemostatic emergency surgery, tube thoracostomy and emergency orthopaedic procedures was predicted with a good accuracy by the NACA score. These procedures were uncommon in our material, however, and our findings in this respect should therefore be interpreted with caution.

The experience of the physician performing the scoring and the time when it is done is important. Alessandrini et al. recently documented a variation in NACA score assigned to the same patient depending on when the scoring took place.^11^ In our material, all patients were scored at the time of maximum severity during the pre-hospital phase, in line with the recommendations suggested by Alessandrini et al.^11^ The rural service studied herein has characteristically long pre-hospital transport times, and subsequently, the scoring took place after a long observation period compared with urban services. More time to observe the patient before scoring might theoretically have given a score more closely correlating with the severity of the patient's condition as determined after hospital admission. This might contribute to the high correlation between NACA score, mortality and need for ventilatory therapy in our study.

The physicians in our service are mainly experienced anaesthesiologists; the majority are specialists in anaesthesiology with more than 2 years of experience in pre-hospital emergency medicine. The experience and training level of the doctors that use the scoring system increases scoring quality according to a study by Knapp et al.^12^ A study from the German ADAC Luftrettung service described the use of the NACA score as highly subjective and showed that even unequivocal conditions such as resuscitation and death, and clearly life-threatening diseases such as acute myocardial infarction, were all often found to be scored incorrectly.^13^

The good predictive ability of the NACA score for mortality and other selected indicators of patient severity is an important finding for the 25 years history of using the NACA score in the National Norwegian air ambulance service. Another important aspect, however, is what purpose the scoring should serve. In the Austrian helicopter emergency medical system (HEMS) system, the NACA score has traditionally been applied in retrospective economical validation of the dispatch, as only patients with NACA score of 4 or more should have access to pre-hospital emergency medicine physicians.^13^ Reimbursement is not an issue in the Norwegian setting, as the National Air Ambulance Services of Norway is an integrated part of the public funded Norwegian health-care system. Furthermore, the NACA score has a limited correlation to more complex patient scoring systems,^2^ such as the Injury Severity Score,^14^ but the NACA scoring's simplicity may explain a far more widespread application in pre-hospital health care than more complex scores, which often rely on data known only after many hours in hospital.

The ability of a simple scoring system to predict correct dispatch in time critical situations seems unlikely to us, and this use of the NACA score has been discouraged by some authors.^2^,^11^,^15^ The decision to dispatch advanced resources must be taken at an early time point, often with limited information about the patient's developing condition. Thus, advanced services must expect an over-triage in order to avoid that the advanced service arrives too late to influence patient outcome. Unfortunately, there is at present no reliable predictor that discriminates patients that will benefit from advanced EMS, such as physician-manned HEMS, from other patients.^16^

If the NACA score is only of limited usefulness for the purposes listed earlier, as well as for quality control and epidemiological studies,^2^,^13^,^15^ why do we use it at all? The only justification for the score is that it is simple, easy to use and permits a comparison of case severity between different services. This justification depends, however, on the scoring system displaying a reasonable reproducibility that it predicts patient outcome well and that it has an acceptable physician to physician variability in a given service. If these indicators of scoring quality are present in each air ambulance base, we believe that the NACA score can be justified for use within a rather homogenous health-care system, such as the National Norwegian air ambulance service.

Even with the strong correlation to important outcome measures described in the present study, use of the NACA data should be limited to purely descriptive purposes. We found high NPVs and low PPVs, which is a result of the low mortality rate in the present study population. This is a factor that limits the use of the score in a clinical setting.

Modifications to the NACA scoring system to include vital parameters have been suggested in order to increase the informative value of the NACA score.^11^,^17^ Whether the NACA score could be modified to perform better in our setting is beyond the scope of the present study.

The shortcomings of the NACA score discussed earlier must be clear to all that apply the data for organisational purposes. The timing of the scoring, appropriate training and a continual audit of scoring, as well as defined purposes for the NACA score, could probably all further improve the validity of the score in a given pre-hospital system.

### Limitations

Unfortunately, 170 patients in the present series were lost to follow-up. This comprises nearly 10% of the patients eligible for inclusion. We cannot formally exclude that this may have affected our results. However, we believe that it is unlikely that the excluded patients have introduced any significant bias as the majority were excluded due to incomplete records. It is plausible that the excluded patients had a mortality and frequency of emergency procedures similar to the included group.

In conclusion, the NACA score predicts mortality and the need for advanced interventions in hospital with reasonable accuracy in our service. It can be used to evaluate the patient material generally in an emergency medical system but not during clinical decision-making in the pre-hospital setting.
